# Mogroside-Rich Extract From *Siraitia grosvenorii* Fruits Ameliorates High-Fat Diet-Induced Obesity Associated With the Modulation of Gut Microbiota in Mice

**DOI:** 10.3389/fnut.2022.870394

**Published:** 2022-06-13

**Authors:** Siyuan Wang, Kexin Cui, Jiahao Liu, Jiahao Hu, Ke Yan, Peng Xiao, Yangqing Lu, Xiaogan Yang, Xingwei Liang

**Affiliations:** ^1^State Key Laboratory for Conservation and Utilization of Subtropical Agro-Bioresources, Guangxi University, Nanning, China; ^2^College of Animal Science and Technology, Guangxi University, Nanning, China

**Keywords:** *Siraitia grosvenorii*, mogroside, obesity, high fat diet, gut microbiota

## Abstract

*Siraitia grosvenorii* is a kind of medicinal food plant. The mogroside-rich extract (MGE) of its fruits can effectively ameliorate obesity, but the underlying mechanisms remain underexplored. In this study, we aimed to determine whether MGE can ameliorate obesity by protecting against the divergences of gut microbiota. Mice were challenged with a high-fat diet (HFD) and treated with MGE by oral gavage. Then, the characteristics of the gut microbiota were determined by 16S rDNA analysis. Our findings showed that MGE could significantly reduce body weight gain and fat tissue weight of the mice fed with HFD. Moreover, MGE markedly attenuated fatty liver, and improved glucose tolerance and insulin sensitivity. We further found that the gut microbiota structures were disturbed by HFD feeding. In particular, the abundance of *Firmicutes* was increased and the abundance of *Bacteroidetes* was decreased, resulting in an increased proportion of *Firmicutes* to *Bacteroidetes* (F/B), which contributes to obesity. Interestingly, the abnormal proportion of F/B of HFD feeding mice was restored to the level of control mice by MGE treatment. Additionally, the abundances of obesogenic microbiota, such as *Ruminiclostridium* and *Oscillibacter* were also decreased after MGE treatment. In summary, our findings demonstrate that MGE can modulate gut microbiota in obese mice and shed new light on how it alleviates obesity.

## Introduction

Obesity is a worldwide pandemic in modern society. It increases the risk of various health problems and has become a leading contributor to diabetes mellitus, cardiovascular diseases, and cancers (1). Given the detrimental effects of obesity on human health, diverse strategies, including surgical operation (2), medicines (3), Chinese medicine conditioning, acupuncture (4, 5), exercise and diet, have been developed to control body weight gain or alleviate obesity (6). Among them, plant extracts with medical and edible properties are receiving increasing attention because they are characterized by effectiveness, safety, and pleasant properties (7, 8).

*Siraitia grosvenorii* (Luo han guo) is a kind of medicinal food plant primarily grown in South China (9, 10). Mogrosides (MGs), the major bioactive components of *S. grosvenorii* fruit, have rich medicinal efficiency, namely, anti-inflammation (11, 12), anti-oxidative stress (13, 14), anticancer (15), neuroprotective (16), and promoting reproduction (17–19). For protecting against obesity, *in vitro* studies have shown that mogrol inhibited adipogenesis in the 3T3L cell line by activating AMP-activated protein kinase (AMPK) activity (20). *In vivo* studies have shown that MGs can reduce body weight gain and attenuate non-alcoholic fatty liver disease (NAFLD) by enhancing the phosphorylation levels of AMPK in the livers of mice challenged with a high-fat diet (HFD) (21). Similarly, Liu et al. reported that mogroside-rich extract (MGE) can alleviate hyperglycemic and hyperlipidemic syndromes in HFD/streptozotocin-induced diabetic mice, in part due to the improvement of insulin sensitivity and activation of hepatic AMPK signaling (22). Although the effects of MGE on attenuating obesity were widely explored, the underlying mechanisms still have not been sufficiently elucidated.

Microbes settle in the gut and play fundamental roles in maintaining host health. In contrast, intestinal dysbacteriosis is closely linked to a variety of health problems, particularly the pathophysiology of obesity (23). It has been revealed that not only between genetically obese mice and their lean littermates but also between obese and lean human volunteers, gut microbial diversity and gut microbiota compositions are changed, especially the relative abundance of *Bacteroidetes* and *Firmicutes*, which are decreased and increased, respectively, in obese individuals (24, 25). Conversely, shaping gut microbiota by colonizing “lean microbiota” or eating certain food can alleviate obesity and metabolic disorders (26–29).

In this study, we speculated that MGs might alleviate obesity associated with the modulation of gut microbiota. To test this hypothesis, male mice were challenged with an HFD to establish a diet-induced obese (DIO) animal model, concomitantly orally administered MGE to determine the effects on alleviating obese phenotypes. Additionally, gut microbial diversities and compositions were characterized to determine how MGE modulates gut microbiota. This study can help us to extend the current understanding of how MGs alleviate obesity.

## Materials and Methods

### Ethical Approval and Animals

The animal experiments were approved by the Institutional Animal Care and Use Committee (IACUC) of Guangxi University and were conducted in accordance with the animal welfare and ethical rules. Three-week-old male C57BL/6 mice were purchased from Beijing Vital River Laboratory Animal Technology Company (Beijing, China). Mice were maintained in individually ventilated cages (IVCs) under 12 h light/12 h dark cycles at an ambient temperature of 22 ± 2°. Mice were *ad libitum* accessed to food and water.

### Treatments

After acclimating for one week, the mice were randomly divided into four groups with eight mice each in group (control, HFD, HFD + MGE300, and HFD + MGE600). The control mice were fed with a chow diet (10% energy from fat, D12450, Research Diets, New Brunswick, NJ) and the other mice were challenged with an HFD (60% energy from fat, D12492, Research Diets). Mice in the control and HFD groups were given orally 100 μl water at 9:00 a.m., while the HFD + MGE300 and HFD + MGE600 mice were orally administered 300 mg/kg or 600 mg/kg body weight MGE, respectively. During treatment for 18 weeks, the body weight and food intake were recorded weekly during treatment.

TheMGE was provided by Layn Biotech, Inc. (Guilin, China). Its main constituents were described in our previous publication (14).

### Body Fat Percentage Measurement

The body fat percentage of mice was assayed by using Small Animal Body Composition Analysis and Imaging System NMR Analyzer (MesoQMR23-060H, Niumag Corporation, Shanghai, China).

### Glucose Tolerance Test and Insulin Tolerance Test

Mice were subjected to Glucose Tolerance Test (GTT) (at 19 weeks of age) and Insulin Tolerance Test (ITT) (at 20 weeks of age) as previously described (30). Briefly, mice were fasted for 12 h or 6 h and then intraperitoneally injected with 2 g/kg body weight D-glucose or 1 IU/kg body weight insulin for GTT and ITT, respectively. Blood was collected from the tail tip at 0, 15, 30, 60, and 120 min post-injection. Glucose concentration was measured using an Accu-Chek Performa (Roche Diagnostics, United States).

### Liver Triglyceride Content Assay

The triglyceride (TG) content in the liver was measured using a TG colorimetric assay kit (Applygen Technologies, Beijing, China) according to the manufacturer’s instructions. Fifty milligrams of liver tissue were grounded in liquid nitrogen, and then TG was extracted. TG concentration was measured using a BioTek Epoch full-wavelength microplate reader (Epoch, United States) at a 550 nm wavelength.

### Hematoxylin and Eosin Staining

Fat and liver tissues were fixed in 4% paraformaldehyde (PFA) overnight at 4°. The samples were embedded in paraffin and cut into 5 μm thick sections according to routine procedures. Hematoxylin and Eosin (H&E) staining was performed according to a standard procedure. Images were captured under a light microscope (Nikon, Eclipse 50i).

### Colonic Microbiota Analyses

The structures of the colonic microbiota were assessed by 16S rRNA amplicon gene sequencing, which was performed by Biomarker Technologies (Beijing, China). Samples were collected for the colons and total genomic DNA was extracted by using the TIANamp Stool DNA Kit (TIANGEN Biotech, Beijing, China), and the V3-V4 regions of the 16S rRNA gene were amplified by using the forward primer 338F (5′-ACTCCTACGGGAGGCAGCA-3′) and reverse primer 806R (5′-GGACTACHVGGGTWTCTAAT-3′). The operational taxonomic unit (OTU) table and taxonomic analysis were obtained from Qiime2 (2019.10) software (31).

### Statistical Analyses

*Post hoc* analyses were performed using Prism 7 software (GraphPad, San Diego, CA, United States) when significance was achieved in the main or simple effects model. Multiple comparisons were corrected using Tukey–Kramer or Dunnett–Hsu adjustment in mixed model procedures. Data are presented as means ± SEM. *P* < 0.05 was considered statistically significant for a null hypothesis of no difference between study test meals. Bioinformatics analyses were carried out at the Multifunction Computer Center of Guangxi University and the R platform (version 4.0.3).

## Results

### Mogroside-Rich Extract Attenuates Body Weight Gain and Fat Accumulation in Diet-Induced Obese Mice

**FIGURE 1:**
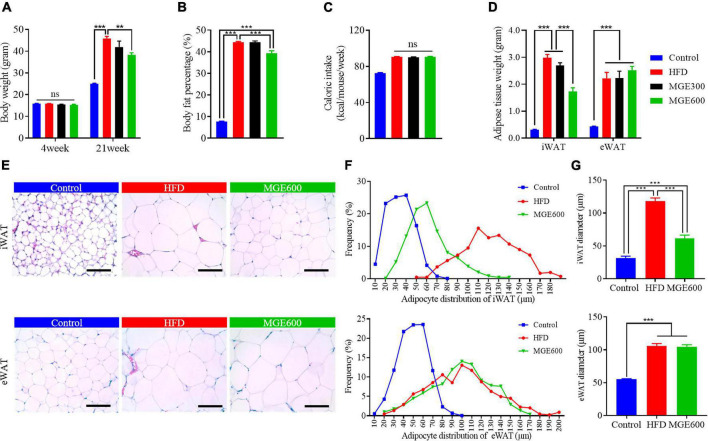
MGE attenuates body weight gain and reduces fat mass in DIO mice. Diet-induced obese (DIO) mice were orally administered 300 and 600 mg body weight MGE from 4 to 21 weeks of age. **(A)** Body weight at the beginning and end of the experiment (*n* = 12). **(B)** Body fat percentage at the end of the experiment (*n* = 8). **(C)** Weekly average calorie intake per mice. **(D)** Inguinal adipose tissue (iWAT) and epididymal adipose tissue (eWAT) weight (*n* = 6). **(E–G)** Representative images of H&E staining, adipocyte distribution, and average adipocyte diameter of iWAT and eWAT (*n* = 6). Scale bar = 100 μm; magnification 20×. **P < 0.01, ***P < 0.001.

After challenge with a HFD for eighteen weeks, the mice had vastly higher body weights (Con vs. HFD; 25.1 ± 0.15 vs. 45.9 ± 0.95 g; *P* < 0.0001) and body fat percentage (Con vs. HFD; 7.7 ± 0.25 vs. 44.4 ± 0.41%; *P* < 0.0001) than the mice fed the control diet. There were no significant differences in body weight or body fat content between mice in the HFD and HFD + MGE300 groups, indicating that 300 mg/kg MGE does not attenuate obesity. However, when the mice were treated with 600 mg/kg MGE, the body weight and body fat content were significantly reduced compared with those of the HFD group (Figures 1A,B), indicating that 600 mg/kg MGE can attenuate HFD-induced obesity. In addition, compared with the control group, the high-fat diet increased the average weekly calorie intake of mice. However, there was no difference in calorie intake between MGE treated group (MGE300, MGE600) and the HFD group (Figure 1C). For individual adipose tissue, when mice were fed an HFD, the inguinal white adipose tissue (iWAT) and epididymal white adipose tissue (eWAT) weights were notably increased compared with those of the mice fed a control diet. A 300 mg/kg MGE did not decrease the iWAT or eWAT weight in the mice fed an HFD. However, 600 mg/kg MGE significantly reduced iWAT weight but had no impact on eWAT weight in the mice fed a HFD (Figure 1D). Consistently, the HFD challenge obviously increased the adipocyte size of iWAT and eWAT in the mice. Interestingly, 600 mg/kg MGE treatment reduced the adipocyte size of iWAT but did not change the adipocyte size of eWAT in DIO mice (Figures 1E–G). Considering the effects of 600 mg/kg MGE on alleviating obesity, this dose was used in the subsequent analyses.

### Mogroside-Rich Extract Reduces Liver Fat Accumulation and Improves Glucose and Insulin Sensitivity in Diet-Induced Obese Mice

**FIGURE 2:**
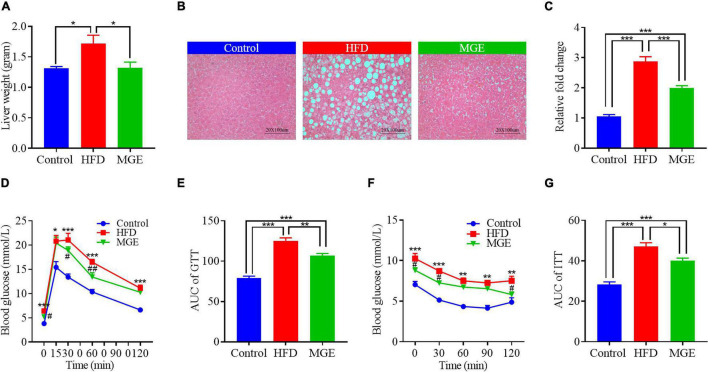
MGE reduces liver fat accumulation and improves glucose and insulin sensitivity in DIO mice. **(A)** Liver weight of mice (*n* = 7). **(B)** Representative images of liver tissue H&E staining. Scale bar = 100 μm. **(C)** Liver triglyceride concentration relative to the control (*n* = 7). **(D,E)** Glucose tolerance test (GTT; *n* = 6). **(F,G)** Insulin tolerance test (ITT; *n* = 5). AUC, area under curve. *P < 0.05, **P < 0.01, ***P < 0.001; ^#^*P* < 0.05, ^##^*P* < 0.01 vs. HFD group.

We next determined whether MGE treatment might ameliorate fatty liver in DIO mice. As shown in Figure 2A, the mice in the HFD group had higher liver weights than those in the control group (Con vs HFD; 1.3 ± 0.03 vs 1.7 ± 0.13 g; *P* < 0.05). H&E staining showed that there were many lipid droplets distributed in the liver of HFD-challenged mice (Figure 2B), suggesting that HFD feeding results in fatty liver. We further assayed TG content in the liver and found that the mice in the HFD group had significantly higher TG content than the mice in the control group (Figure 2C). This further confirmed that the HFD challenge leads to fatty liver in mice. As expected, MGE treatment markedly reduced the liver weight and TG content in the livers of mice challenged with an HFD, indicating that MGE can alleviate fatty liver in DIO mice (Figures 2A–C).

Compared with the control group, the HFD group had significantly higher AUCs of GTT and ITT (Figures 2D–G), indicating that the HFD challenge reduces glucose and insulin sensitivity in mice. However, MGE treatment notably reduced the AUCs of the GTT and ITT in the mice challenged with a HFD (Figures 2D–G). Taken together, MGE supplementation alleviated fatty liver and improved global metabolic capacities in DIO mice.

### Effects of Treatment on Intestinal Microbiota α-Diversity and β-Diversity

High-throughput sequencing generated 1,919,310 raw reads. After removing the low-quality sequences, there were 1,787,645 clean tags, and each sample produced an average of 74,485 clean tags. Based on a 97% similarity cutoff value, all the effective reads were clustered into operational taxonomic units (OTUs).

**FIGURE 3:**
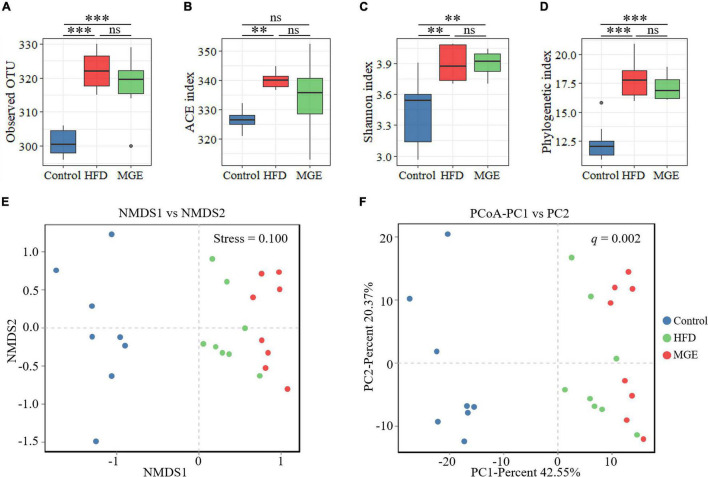
Effects of MGE treatment on intestinal microbiota α-diversity and β-diversity. **(A)** Observed OTU in each group. **(B)** Abundance-based coverage estimator (ACE) index. **(C)** Shannon index. **(D)** Phylogenetic diversity (PD). **(E,F)** Beta diversity analysis: **(E)** non-metric multidimensional scaling (NMDS), **(F)** principal coordinates analysis (PCoA) of the unweighted UniFrac distance matrix. Group significance was statistically analyzed by using the PERMANOVA method. **P < 0.01, ***P < 0.001.

To identify the differences in intestinal microbiota structure among groups, the α-diversity and β-diversity were compared. As shown in Figures 3A–D, the α-diversity indexes (ACE, Shannon, and phylogenetic diversity) in the HFD group were significantly increased compared with those in the control group, but there were no differences between the HFD and MGE groups. On the other hand, non-metric multidimensional scaling (NMDS) and principal coordinates analysis (PCoA) of the unweighted UniFrac distance matrix were used to identify β-diversity. As shown in Figures 3E,F, NMDS and PCoA showed that the clusters of the HFD group were significantly different from those in the control group, but MGE treatment significantly ameliorated these alters. Taken together, the above results indicate that MGE can restore the changes of community structure in microbiota but not species types in DIO mice.

### Mogroside-Rich Extract Restores the Relative Abundance of *Firmicutes* and *Bacteroidetes* in Diet-Induced Obese Mice

**FIGURE 4:**
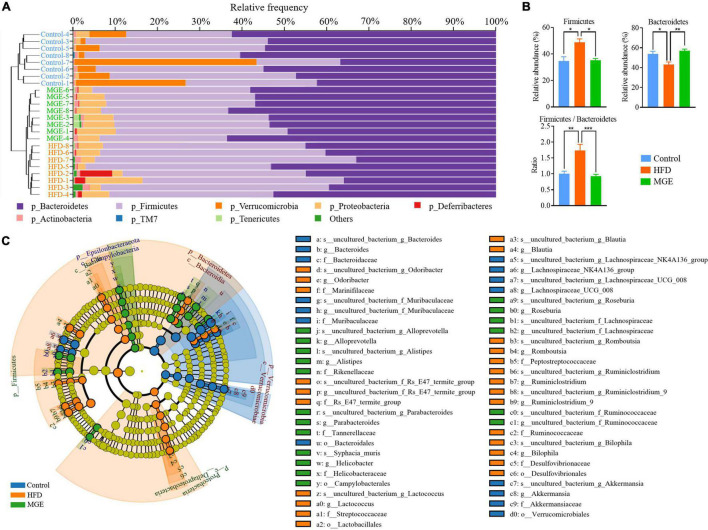
MGE restores the relative abundance of *Firmicutes* and *Bacteroidetes* in DIO mice. **(A)** Microbiota taxonomic composition clustering histogram at the phylum level; left: unweighted pair-group method with arithmetic mean (UPGMA) tree; right: histogram of microbiota abundance. **(B)** Relative abundance of *Firmicutes* and *Bacteroidetes* and the ratio of *Firmicutes* to *Bacteroidetes*. **(C)** Taxonomic cladogram obtained from linear discriminant analysis effect size (LEfSe) sequence analysis. Species taxonomy from phyla to genus (inside-outside). The diameter of each circle represents the relative abundance of the taxon, and the color corresponds to the grouping. Undifferentiated microbiota components (yellow). The predominant microbiota component at each taxonomic level is represented by different colors. *P < 0.05, **P < 0.01, ***P < 0.001.

Combining the unweighted pair-group method with arithmetic mean (UPGMA), the relative abundance of the gut microbiota at the phylum level was identified by QIIME2 taxonomic analysis. As shown in Figure 4A, the HFD group had an apparent difference in taxonomic composition compared with the control group, but there was an apparent similarity between the MGE group and the control group. For individual phyla, the relative abundance of *Firmicutes* was significantly increased, but that of *Bacteroidetes* was significantly decreased due to HFD feeding, resulting in an increase in the ratio of *Firmicutes* to *Bacteroidetes* (F/B) in DIO mice (Figure 4B). However, MGE treatment restored the relative abundance of *Firmicutes* and *Bacteroidetes*, as well as the ratio of F/B to control levels, in DIO mice (Figure 4B). In addition, the linear discriminant analysis effect size (LEfSe) method was used to further determine differences in microbiota abundances (Figure 4C). There was 67 differentially abundant microbiota, and 49 out of 67 (73.1%) belonged to *Bacteroidetes* and *Firmicutes*, which further confirmed that MGE can restore the abundance of *Firmicutes* and *Bacteroidetes* in DIO mice.

### Mogroside-Rich Extract Restores the Abundance of Intestinal Microbiota at the Genus Level in Diet-Induced Obese Mice

**FIGURE 5:**
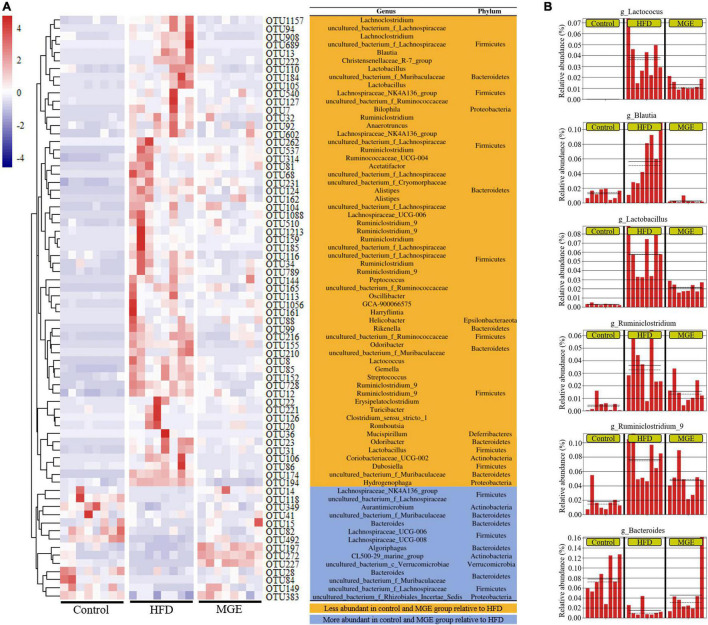
MGE restores the abundance of intestinal microbiota at the genus level in DIO mice. **(A)** OTU differential clustering heatmap with representative bacterial taxon information (genus and phylum). **(B)** The relative abundance of bacterial genera significantly recovered by MGE treatment (*P* < 0.01); solid and dashed lines indicate the mean and median, respectively.

To further explore the effect of MGE on bacterial taxonomic composition at the genus level, a clustering heatmap was constructed. Figure 5A displays the top 72 OTUs with significant differences (*P* < 0.01) selected for clustering analysis. Fifty-eight out of 72 OTUs were significantly less abundant in the control group and MGE group than in the HFD group. Interestingly, *Lactococcus*, *Blautia*, *Lactobacillus*, *Ruminiclostridium_9*, *Oscillibacter*, and *Ruminiclostridium* belong to *Firmicutes* and are obesogenic microbiota (Figure 5B). In contrast, 14 out of 72 OTUs, including *Bacteroides*, were significantly more abundant in the control group and MGE group than in the HFD group (Figure 5B). Taken together, MGE can restore the intestinal microbiota at the genus level in DIO mice.

### Correlation Analyses of Gut Microbiota Structures and Obese Phenotypes

**FIGURE 6:**
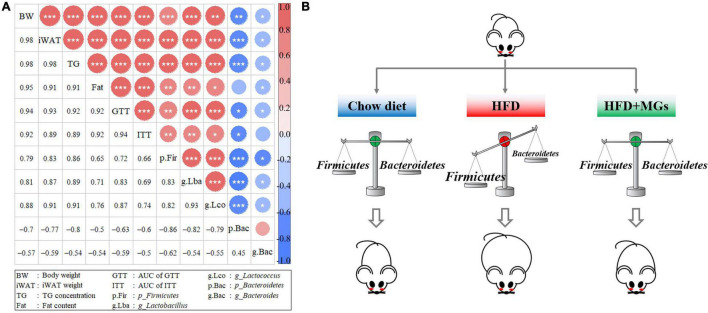
Correlation analyses of gut microbiota structures and obese phenotypes. **(A)** The diagonals represent the indicator; positive correlation (red), negative correlation (blue); the circle diameters correspond to the absolute value of correlation coefficients; the asterisk corresponds to significance: *P < 0.05, **P < 0.01, ***P < 0.001. **(B)** The cartoon represents how MGs attenuate obesity by shaping gut microbiota.

We further performed a correlation analysis between the gut microbiota structures and obese phenotypes. Based on the Spearman index, we found that the abundances of *Firmicutes*, *Lactobacillus*, and *Lactococcus* were positively correlated with obese phenotypes, such as body weight, iWAT weight, body fat content, liver TG concentration, and AUC of GTT and ITT. However, the abundances of *Bacteroidetes* and *Bacteroides* were negatively correlated with the above obesity parameters (Figure 6A). The results of correlation analysis show that MGs alleviate obesity and maybe associated with the gut microbiota modulation (Figure 6B).

## Discussion

Previous studies have demonstrated that MGs can attenuate obesity, but their effect on modulating gut microbiota in this condition remains unknown. In this study, we first observed that MGE treatment can alleviate obesity by inhibiting body weight gain and fat accumulation in adipose tissues and liver, improving insulin and glucose sensibilities in DIO mice. Importantly, we further found that MGE treatment modulates gut microbiota, particularly restores the ratio of *Firmicutes* to *Bacteroidetes* and reduces the relative abundance of obesogenic microbiota. Our findings depict that MGE attenuates obesity at least in part by gut microbiota modulation.

First, we used a DIO mouse model to determine the beneficial effects of MGs on obese animals. When the mice were fed an HFD from 4 to 21 weeks of age, the body weight, global fat mass, and adipose tissue weight were vastly increased. In addition, the increased adipocyte size and accumulated fat content in the liver indicated that the HFD challenge induced hypertrophic adipose tissues and NAFLD, respectively. Moreover, HFD feeding also impairs global metabolic capacities by reducing glucose and insulin sensibilities. Combined with previous reports (32–35), all the above defective parameters demonstrated that a DIO animal model was successfully established in this study. As expected, when DIO mice were orally administered MGE, body weight gain and fat accumulation were attenuated, and global metabolic capacities were also improved. Our findings reconfirmed that MGs can alleviate obesity (21, 22, 36, 37); therefore, this model can be used to explore the modulation of gut microbiota by MGE.

We next used 16S sequencing to investigate how MGE affects gut microbial compositions and diversities in DIO mice. α-Diversity and β-diversity are two key indicators that reflect the differences in gut microbial communities (38, 39). We found that the α-diversity of the gut microbiota was increased in DIO mice but was not altered by MGE treatment. Similarly, the β-diversity of the gut microbiota was vastly different between the control and HFD groups. Interestingly, MGE treatment could partly restore this alteration in DIO mice. Consistently, a previous study showed that MG can alleviate metabolic disorders by regulating the gut microbiota in T2DM rats (40). This study and a previous report (40) show that MGs can regulate gut microbiota communities, which is a potential mechanism by which MGs attenuate metabolic disorders.

We further found that the abundance of *Firmicutes* was increased while the abundance of *Bacteroidetes* was decreased in DIO mice, whereas these changes were recovered by MGE treatment. It has been revealed that *Firmicutes* are positively associated with the pathogenesis of obesity; in contrast, *Bacteroidetes* exhibit anti-obesity activity (41). Moreover, the abundance of *Firmicutes* is increased and that of *Bacteroidetes* is reduced in obese individuals (42). Thus, on the one hand, the reduction of *Firmicutes* abundance by antibiotics (vancomycin and bacitracin) can improve insulin resistance in DIO mice (43). On the other hand, *Bacteroidetes* abundance is significantly decreased in leptin-deficient obese mice compared with lean mice (44) because *Bacteroidetes* can obtain energy from indigestible polysaccharides and produce SCFAs to regulate energy metabolism (45). Therefore, MGs attenuate obesity was associated with the intestinal microbiota of specific *Firmicutes* and *Bacteroidetes* at the phylum level.

We next deeply explored the effects of MGE on the intestinal microbiota at the genus level in DIO mice. We observed decreased abundances of *Bacteroides* and increased abundances of *Lactobacillus*, *Lactococcus*, *Ruminiclostridium*, *Ruminiclostridium_9*, *Oscillibacter*, and *Blautia* in DIO mice. Interestingly, these changes were effectively restored by MGE treatment. Consistently, germ-free mice colonized with gut microbiota from DIO mice showed NAFLD, suggesting that certain gut microbiota, such as the genus *Bacteroides*, are responsible for hepatic lipid accumulation (46–48). In addition, *Lactobacillus* was positively correlated with insulin resistance and body weight gain. Furthermore, studies have revealed that *Lactococcus*, *Ruminiclostridium*, *Ruminiclostridium_9*, *Oscillibacter* and *Blautia* are associated with obesity in animal models and humans (49–51). Furthermore, our correlation analyses show that HFD-mediated gut microbiota abundance changes are significantly related to obesity phenotypes, e.g., body weight gain, fat mass, and insulin sensitivity.

## Conclusion

*Siraitia grosvenorii* is a kind of medicinal food plant, and its fruit extracts have anti-obesity effects. Our findings reveal that MGE can alleviate intestinal dysbacteriosis in obese mice. Of note, fecal microbiota transplantation (FMT) or co-housing experiments should further strengthen the conclusion that MGE alleviates obesity by gut microbiota modulation.

## Data Availability Statement

The datasets presented in this study can be found in online repositories. The name of the repository and accession number can be found below: National Center for Biotechnology Information (NCBI) BioProject, https://www.ncbi.nlm.nih.gov/bioproject/, PRJNA811511.

## Ethics Statement

The animal study was reviewed and approved by Institutional Animal Care and Use Committee (IACUC) of Guangxi University.

## Author Contributions

SW, KC, and XL conceived the study. SW, KC, JL, JH, KY, and PX performed the experiments. SW, XY, YL, and XL analyzed the data and wrote the manuscript. All authors reviewed the manuscript and approved the final version of the manuscript.

## Conflict of Interest

The authors declare that the research was conducted in the absence of any commercial or financial relationships that could be construed as a potential conflict of interest.

## Publisher’s Note

All claims expressed in this article are solely those of the authors and do not necessarily represent those of their affiliated organizations, or those of the publisher, the editors and the reviewers. Any product that may be evaluated in this article, or claim that may be made by its manufacturer, is not guaranteed or endorsed by the publisher.
